# PTBP1 variants displaying altered nucleocytoplasmic distribution are responsible for a neurodevelopmental disorder with skeletal dysplasia

**DOI:** 10.1172/JCI182100

**Published:** 2025-09-18

**Authors:** Aymeric Masson, Julien Paccaud, Martina Orefice, Estelle Colin, Outi Mäkitie, Valérie Cormier-Daire, Raissa Relator, Sourav Ghosh, Jean-Marc Strub, Christine Schaeffer-Reiss, Carlo Marcelis, David A. Koolen, Rolph Pfundt, Elke de Boer, Lisenka E.L.M. Vissers, Thatjana Gardeitchik, Lonneke A.M. Aarts, Tuula Rinne, Paulien A. Terhal, Nienke E. Verbeek, Linda C. Zuurbier, Astrid S. Plomp, Marja W. Wessels, Stella A. de Man, Arjan Bouman, Lynne M. Bird, Reem Saadeh-Haddad, Maria J. Guillen Sacoto, Richard Person, Catherine Gooch, Anna C.E. Hurst, Michelle L. Thompson, Susan M. Hiatt, Rebecca O. Littlejohn, Elizabeth R. Roeder, Mari Mori, Scott E. Hickey, Jesse M. Hunter, Kristy Lee, Khaled Osman, Rana Halloun, Ruxandra Bachmann-Gagescu, Anita Rauch, Dagmar Wieczorek, Konrad Platzer, Johannes Luppe, Laurence Duplomb-Jego, Fatima El It, Yannis Duffourd, Frédéric Tran Mau-Them, Celine Huber, Christopher T. Gordon, Fulya Taylan, Riikka E. Mäkitie, Alice Costantini, Helena Valta, Stephen Robertson, Gemma Poke, Michel Francoise, Andrea Ciolfi, Marco Tartaglia, Nina Ekhilevitch, Rinat Zaid, Michael A. Levy, Jennifer Kerkhof, Haley McConkey, Julian Delanne, Martin Chevarin, Valentin Vautrot, Valentin Bourgeois, Sylvie Nguyen, Nathalie Marle, Patrick Callier, Hana Safraou, Angela Morgan, David J. Amor, Michael S. Hildebrand, David Coman, Marion Aubert Mucca, Julien Thevenon, Fanny Laffargue, Frédéric Bilan, Céline Pebrel-Richard, Grace Yoon, Michelle M. Axford, Luis A. Pérez-Jurado, Marta Sevilla-Porras, Douglas L. Black, Christophe Philippe, Bekim Sadikovic, Christel Thauvin-Robinet, Laurence Olivier-Faivre, Michela Ori, Quentin Thomas, Antonio Vitobello

**Affiliations:** 1 Université Bourgogne Europe, CHU Dijon Bourgogne, Centre de recherche Translationnelle en Médecine moléculaire – Inserm UMR1231 équipe GAD, Dijon, France.; 2 Department of Biology, University of Pisa, Pisa, Italy.; 3 University Hospital of Angers, Angers, France.; 4 Children’s Hospital, Pediatric Research Center, University of Helsinki and Helsinki University Hospital, Helsinki, Finland.; 5 Department of Molecular Medicine and Surgery, Karolinska Institutet, Stockholm, Sweden.; 6 Clinical Genetics and Genomics, Karolinska University Hospital, Solna, Sweden.; 7 Folkhälsan Institute of Genetics, Helsinki, Finland.; 8 Research Program for Clinical and Molecular Metabolism, Faculty of Medicine, University of Helsinki, Helsinki, Finland.; 9 INSERM UMR1163, Imagine Institute, Paris Cité University, Paris, France.; 10 Service de médecine génomique des maladies rares, Centre de référence pour les maladies osseuses constitutionnelles, AP-HP, Hôpital Necker-Enfants Malades, Paris, F-75015, France.; 11 Verspeeten Clinical Genome Centre, London Health Sciences, London, Ontario, Canada.; 12 Department of Pathology and Laboratory Medicine, Western University, London, Ontario, Canada.; 13 Laboratoire de Spectrométrie de Masse Bio Organique, IPHC UMR 7178 CNRS, Université de Strasbourg, Strasbourg, 67087, France.; 14 Infrastructure Nationale de Protéomique ProFI, Strasbourg, 67087, France.; 15 Department of Human Genetics, Donders Institute for Brain, Cognition, and Behavior, Nijmegen, Netherlands.; 16 Department of Human Genetics, Radboud University Medical Center, Nijmegen, Netherlands.; 17 Department of Pediatrics, Amalia Children’s Hospital, Radboud University Medical Center, Nijmegen, Netherlands.; 18 Department of Genetics, University Medical Center Utrecht, Utrecht, Netherlands.; 19 Department of Clinical Genetics, Amsterdam UMC Location Research and Diagnostic Centre ADORE, Amsterdam, Netherlands.; 20 Department of Human Genetics, Amsterdam University Medical Center, University of Amsterdam, Amsterdam, Netherlands.; 21 Department of Clinical Genetics, Erasmus MC University Medical Center, Rotterdam, Netherlands.; 22 Department of Pediatrics, Amphia Hospital, Breda, Netherlands.; 23 Department of Pediatrics, Rady Children’s Hospital San Diego, Univeristy of California, San Diego, California, USA.; 24 Division of Genetics, Department of Pediatrics, Medstar Georgetown University Hospital, Washington, DC, USA.; 25 GeneDx, LLC, Gaithersburg, Maryland, USA.; 26 Division of Genetics and Genomic Medicine, Department of Pediatrics, Washington University School of Medicine, St Louis, Missouri, USA.; 27 Department of Genetics, University of Alabama at Birmingham, Birmingham, Alabama, USA.; 28 HudsonAlpha Institute for Biotechnology, Huntsville, Alabama, USA.; 29 Department of Pediatrics, Baylor College of Medicine, San Antonio, Texas, USA.; 30 Division of Genetic & Genomic Medicine, Nationwide Children’s Hospital, Columbus, Ohio, USA.; 31 The Ohio State University College of Medicine, Columbus, Ohio, USA.; 32 Institute for Genomic Medicine, Nationwide Children’s Hospital, Columbus, Ohio, USA.; 33 Caris Life Sciences, Phoenix, Arizona, USA.; 34 Genetics institute and; 35 Pediatric endocrinology Unit, Ruth Rappaport Children’s Hospital, Rambam Health Care Campus, Haifa, Israel.; 36 Institute of Medical Genetics, University of Zurich, Schlieren, Switzerland.; 37 University Children’s Hospital Zurich, Zurich, Switzerland.; 38 Institute of Human Genetics, Medical Faculty and University Hospital, Heinrich-Heine-University, Düsseldorf, Germany.; 39 Institute of Human Genetics, University of Leipzig Medical Center, Leipzig, Germany.; 40 Université Bourgogne Europe, CHU Dijon Bourgogne, Laboratoire de Génomique Médicale, Centre Neomics, FHU-TRANSLAD, Centre de recherche Translationnelle en Médecine moléculaire – Inserm UMR1231 équipe GAD, Dijon, France.; 41 GCS AURAGEN, Lyon, France.; 42 Department of Otorhinolaryngology, Head and Neck Surgery, Helsinki University Hospital, University of Helsinki, Helsinki, Finland.; 43 Department of Women’s and Children’s Health, Dunedin School of Medicine, University of Otago, Dunedin, New Zealand.; 44 Genetics Health Service New Zealand, Wellington Hospital, Wellington, New Zealand.; 45 Centre Hospitalier William Morey, Chalon-sur-Saône, France.; 46 Molecular Genetics and Functional Genomics and; 47 Molecular Genetics and Functional Genomics, Ospedale Pediatrico Bambino Gesù, IRCCS, Rome, Italy.; 48 The Genetics Institute, Rambam Health Care Campus, Haifa, Israel.; 49 Université Bourgogne Europe, CHU Dijon Bourgogne, Laboratoire de Génétique Chromosomique et Moléculaire, Dijon, France.; 50 Murdoch Children’s Research Institute, Parkville, Australia.; 51 Department of Paediatrics, Royal Children’s Hospital, University of Melbourne, Parkville, Australia.; 52 Department of Medicine, Austin Health, University of Melbourne, Heidelberg, Australia.; 53 Queensland Children’s Hospital, Brisbane, Australia.; 54 School of Medicine, Univesrity of Queensland, Brisbane, Australia.; 55 Service de Génétique Médicale, Hôpital Purpan, CHU, 31059, Toulouse, France.; 56 Service de Génétique, Génomique et Procréation, CHU Grenoble Alpes, Grenoble, France.; 57 Université Grenoble Alpes, INSERM U 1209, CNRS UMR 5309, Institut for Advanced Biosciences, Grenoble, France.; 58 Service de Génétique Médicale, CHU de Clermont-Ferrand, Clermont-Ferrand, France.; 59 CHU de Poitiers, Service de Génétique, EA3808 NEUVACOD, Poitiers, France.; 60 Cytogénétique Médicale, Centre Hospitalier Universitaire de Clermont-Ferrand, CHU Estaing, 63000 Clermont-Ferrand, France.; 61 Divisions of Neurology and Clinical and Metabolic Genetics, Department of Paediatrics, and; 62 Division of Genome Diagnostics, Department of Laboratory Medicine and Pathobiology, The Hospital for Sick Children, University of Toronto, Toronto, Ontario, Canada.; 63 Universitat Pompeu Fabra, CIBERER (Centro de Investigación Biomédica en Red de Enfermedades Raras), Barcelona, Spain.; 64 Hospital del Mar – Hospital del Mar Research Institute, Barcelona, Spain.; 65 Department of Microbiology, Immunology, and Molecular Genetics, Molecular Biology Institute, David Geffen School of Medicine, UCLA, Los Angeles, California, USA.; 66 Université Bourgogne Europe, CHU Dijon Bourgogne, Centre de référence maladies rares déficiences intellectuelles de causes rares, Centre de Génétique, FHU-TRANSLAD, Dijon, France.; 67 Université Bourgogne Europe, CHU Dijon Bourgogne, Centre de Référence maladies rares, Anomalies du Développement et syndromes malformatifs, Centre de Génétique, FHU-TRANSLAD, Dijon, France.

**Keywords:** Development, Genetics, Bone development, Genetic diseases, RNA processing

## Abstract

Polypyrimidine tract-binding protein 1 (PTBP1) is a heterogeneous nuclear ribonucleoprotein primarily known for its alternative splicing activity. It shuttles between the nucleus and cytoplasm via partially overlapping N-terminal nuclear localization (NLS) and export (NES) signals. Despite its fundamental role in cell growth and differentiation, its involvement in human disease remains poorly understood. We identified 27 individuals from 25 families harboring de novo or inherited pathogenic variants — predominantly start-loss (89%) and, to a lesser extent, missense (11%) — affecting NES/NLS motifs. Affected individuals presented with a syndromic neurodevelopmental disorder and variable skeletal dysplasia with disproportionate short stature with short limbs. Intellectual functioning ranged from normal to moderately delayed. Start-loss variants led to translation initiation from an alternative downstream in-frame methionine, resulting in loss of the NES and the first half of the bipartite NLS, and increased cytoplasmic stability. Start-loss and missense variants shared a DNA methylation episignature in peripheral blood and altered nucleocytoplasmic distribution in vitro and in vivo with preferential accumulation in processing bodies, causing aberrant gene expression but normal RNA splicing. Transcriptomic analysis of patient-derived fibroblasts revealed dysregulated pathways involved in osteochondrogenesis and neurodevelopment. Overall, our findings highlight a cytoplasmic role for PTBP1 in RNA stability and disease pathogenesis.

## Abstract 2

Altered nucleocytoplasmic distribution of the RNA-bining protein PTBP1 results in a syndromic neurodevelopmental disorder associated with skeletal dysplasia.

## Graphical abstract

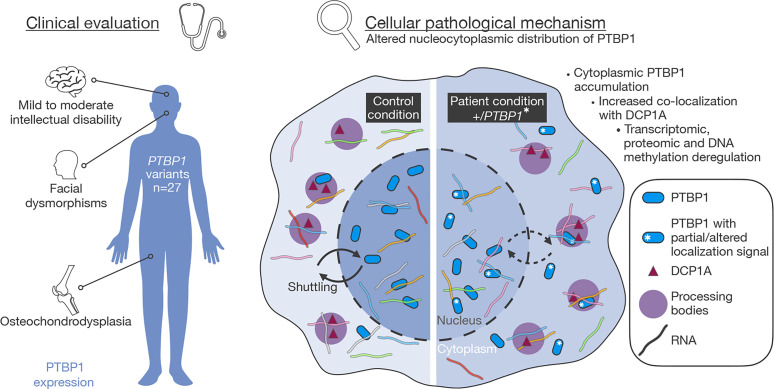

## Introduction

Heterogeneous nuclear ribonucleoproteins (hnRNPs) constitute a large superfamily of RNA-binding proteins (RBPs) involved in several aspects of RNA metabolism, including biogenesis, maturation, transport, localization, storage, translation, and degradation, contributing to a tight regulation of gene expression (1–4). Since hnRNP function is highly dependent on subcellular localization, their distribution in different cell compartments is critical to orchestrate these aspects of RNA metabolism. Most hnRNPs harbor both nuclear localization signals (NLSs) and nuclear export signals (NESs), enabling dynamic nucleocytoplasmic shuttling, although they predominantly localize to the nucleus under steady-state conditions (2, 5–8). Proper subcellular localization is not only essential for RNA metabolism but also underpins cellular processes such as polarity establishment, transcriptional regulation, and translational control (9–12). Dysregulation of hnRNP function has been implicated in a broad spectrum of human diseases, including cancer, neurodevelopmental disorders, and neurodegeneration, making hnRNPs potential therapeutic targets (2, 13–21).

Among hnRNPs, the polypyrimidine tract-binding protein (PTBP) family — comprising PTBP1 (also known as hnRNP I), PTBP2 (nPTB), and PTBP3 (ROD1) — represents structurally related RNA-binding proteins (RBPs) with approximate molecular weights of 57 kDa. These paralogs are encoded by *PTBP1* (MIM 600693), *PTBP2* (MIM 608449), and *PTBP3* (MIM 607527), located on human chromosomes 19, 1, and 9, respectively (22–24). While they are best known for their roles in regulating alternative splicing (25–29), they also modulate a plethora of additional processes depending on their subcellular context, including mRNA polyadenylation, export, localization, stability, translation initiation, and cell cycle regulation (2, 30–36).

PTBP1 and PTBP2 have been more extensively studied. PTBP1 is expressed in many progenitor cells and tissues, while PTBP2 has a more restricted expression, mainly in the brain, where it becomes the predominant PTBP during neural differentiation and maturation, replacing PTBP1 in a tightly regulated developmental switch (37–40).

Structurally, PTBP1 and PTBP2 display high sequence conservation, particularly within their 4 RNA recognition motifs (RRMs), where amino acid (AA) identity exceeds 80% (31, 41). All 3 proteins contain conserved NES and NLS sequences located within the first 50 AAs in the N-terminal end, allowing them to shuttle between the nucleus and cytoplasm (41–45). In PTBP1 and PTBP2, key residues mediating nuclear export map to positions 11–16, with lysine 13 and arginine 14 playing essential roles, and glycine residues at positions 11 and 15 contributing to intermediate activity (45). Phosphorylation of serine 16 by 3′,5′-cAMP–dependent protein kinase A (PKA) has been shown to promote PTBP1 accumulation in the cytoplasm (46).

The NES partially overlaps with a bipartite NLS, with residues 13–14 and 45–48 being especially critical for nuclear import (43, 44). Mutational studies using fused constructs containing the first 60 AAs of PTBP1 linked to reporter proteins like chloramphenicol acetyl transferase (CAT) or green fluorescent protein (GFP) have demonstrated that deletion or mutation of either NLS element results in impaired nuclear targeting and cytoplasmic accumulation (43, 44, 46). These studies underscore the importance of intact localization signals for proper importin-α binding and nuclear pore complex translocation.

The RNA-binding capacity of PTBPs is mediated by their 4 RRMs, separated by flexible linker regions. PTBP1 and PTBP2 undergo alternative splicing to produce isoforms with distinct splicing activities and differential RNA-binding profiles (47–49). PTBP1 has 3 main isoforms, which arise from alternative splicing of exon 9: PTBP1-1 (531 AAs) lacks exon 9 entirely, while PTBP1-2 and PTBP1-4 include 19 and 26 AAs, respectively. Additional isoform diversity arises from variation in 5’ and 3’ untranslated regions (UTRs) (50).

Beyond their nuclear-splicing roles, PTBP1 and PTBP2 also function in the cytoplasm. For instance, they have been shown to promote cap-independent translation via internal ribosome entry site binding, facilitating the expression of target transcripts such as insulin receptor or the P53 mRNAs (33, 34). Furthermore, they stabilize transcripts by binding 3′UTR elements, thereby preventing degradation via nonsense-mediated decay (NMD) (51, 52). PTBP1 also plays a key role in RNA transport and localization. During coronavirus infection, it relocates transiently to the cytoplasm, where it colocalizes with stress granules and modulates posttranscriptional regulation of viral RNA expression colocalizing with T-cell intracellular antigen-1 (TIA-1) and TIA-1-related protein (TIAR) markers (35). In *Xenopus laevis* oocytes, PTBP1 mediates the vegetal localization of Vg1 mRNA, a process essential for early embryonic axis patterning (36).

Despite the established involvement of PTBPs in RNA metabolism, their direct contribution to human genetic disease has remained underexplored. Here, we report on a cohort of 27 individuals harboring start-loss or missense variants in *PTBP1*. Functional analysis of these variants demonstrates that they disrupt normal nucleocytoplasmic localization of the protein. Clinically, affected individuals present with shared phenotypic features, including a neurodevelopmental disorder of variable severity and skeletal dysplasia. Moreover, we identified 2 unrelated individuals with candidate heterozygous de novo variants in *PTBP2* — specifically, a start-loss and a missense change affecting the NES/NLS regions — who exhibited isolated neurodevelopmental phenotypes. These variants were similarly shown to impair nucleocytoplasmic localization, suggesting a convergent pathogenic mechanism between PTBP1 and PTBP2 that merits further investigation.

Collectively, our findings establish a link between PTBP1 dysfunction and developmental disease and suggest that altered PTBP2 localization may contribute to a similar pathomechanism, highlighting the importance of precise subcellular localization in PTBP function.

## Results

### Individuals with variants in PTBP1.

**Figure 1:**
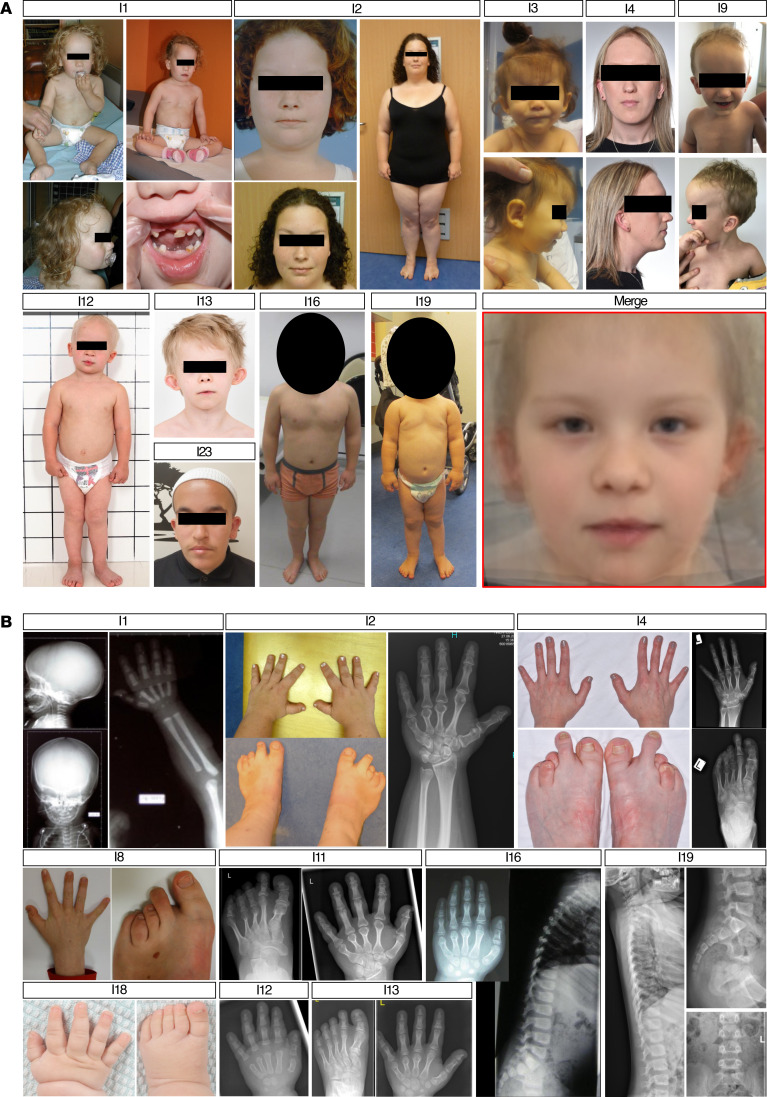
Clinical findings. (**A**) Facial features of individuals with PTBP1 start-loss variants with a computed “average face” (red box “merge”). (**B**) Radiological and clinical skeletal findings. I 1 (5 days old): skull and limb X-rays showing acrocrania and advanced carpal bone maturation. I 2 (19 years old): hand X-ray and clinical images showing brachymetacarpia (except second ray) and brachydactyly (II and III phalanges). I 4 (27 years old): hand and foot images/X-rays showing brachymetacarpia, brachymetatarsia (digits I, III, and IV), brachytelephalangy, brachymesophalangy (digits II, V), and cone-shaped epiphysis (second phalanx, digit II). I 8 (11 years old): hand and foot images showing brachymetacarpia and brachytelephalangy (digits III–V), brachymetatarsia (digits III–V), fifth toe clinodactyly, sandal gap, and hallux valgus. I 11 (24 years old): hand and foot X-rays showing brachymetacarpia, brachytelephalangy, brachymesophalangy (digits II, V), cone-shaped epiphysis (second phalanx, digit II), and brachymetatarsia (digits II, III, V). I 18 (9 months old): hand and foot images showing short hand with brachymetacarpia. I 12 (1 year): hand X-ray showing advanced carpal maturation and cone-shaped distal epiphyses. I 13 (8 years old): hand and foot X-rays showing generalized brachymetacarpia, brachymesophalangy (digits II–V), brachytelephalangy with cone-shaped epiphyses, and brachymetatarsia. I 16 (hand: 7 years old; spine: 2 years old): hand X-ray showing brachymetacarpia, brachytelephalangy, and brachymesophalangy (digits II, V); cone-shaped epiphyses (middle phalanges of digit II, V; distal phalanges of digits I, IV); spine X-ray showing dysplastic lumbar vertebrae with anterior-posterior height disparity. I 19 (3 years old): spine X-ray showing dysplastic vertebral bodies with uneven anterior-posterior height.

**Figure 2:**
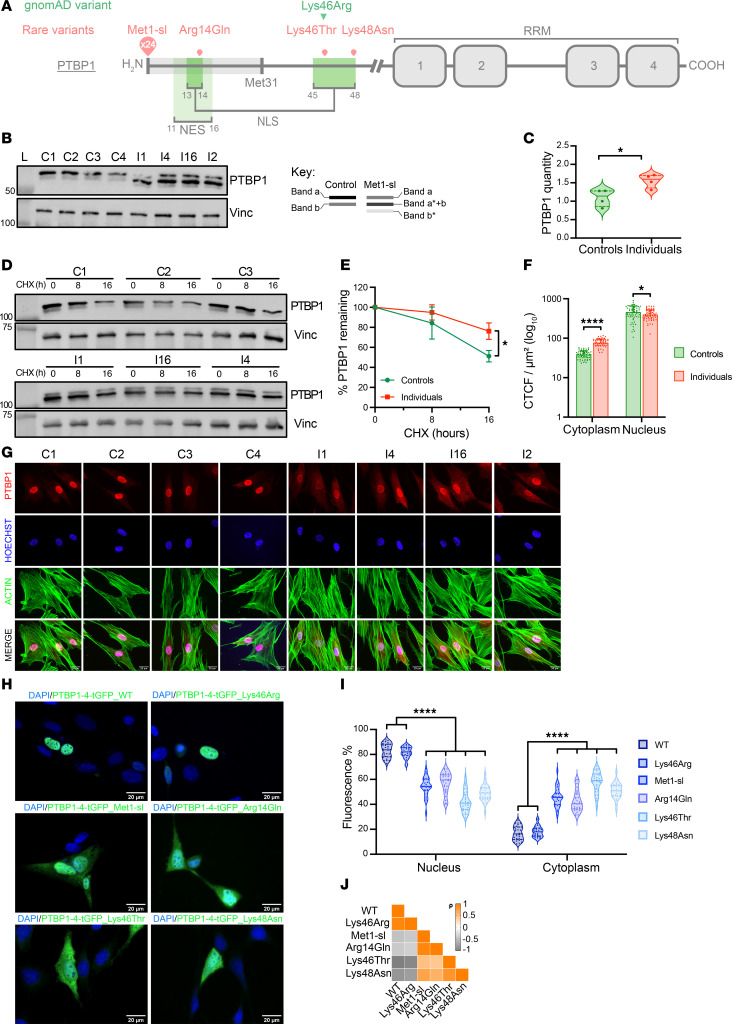
Altered nucleocytoplasmic distribution of PTBP1 mutants. (**A**) PTBP1 variants identified in our cohort (red) and a benign variant (green), mapped to functional domains. The untranslated region in start-loss variants is shaded gray. Schematics are not to scale. (**B**) Western blot of PTBP1 isoforms in fibroblast from controls and start-loss individuals (left); band interpretation shown on the right: band “a” corresponds to overlapping PTBP1-2 and PTBP1-4 isoforms; band “b” to PTBP1-1. Asterisked bands represent start-loss isoforms. Vinculin is the loading control. *n* = 4 independent experiments. (**C**) Quantification of PTBP1 bands from **B**; Mann–Whitney U test. (**D**) Cycloheximide chase assay in fibroblast at 0, 8, or 16 hours. *n* = 3 independent experiments. (**E**) Quantification of PTBP1 expression from **D**; Student’s *t* test. (**F**) Nuclear versus cytoplasmic PTBP1 (red) levels in immunostained fibroblasts shown in **G**; Hoechst (blue), actin (green). Sixty cells per condition were analyzed; Mann–Whitney U test. (**H**) PTBP1-4-tGFP localization (green) in NIH-3T3 cells transfected with WT, start-loss, or missense constructs; DAPI (blue). *n* = 3 independent experiments. (**I**) Quantification of the nuclear/cytoplasmic fluorescence in cells from (**H**). *N* = 30 cells/condition; Mann–Whitney U test. (**J**) Spearman’s correlation analysis of nuclear/cytoplasmic distribution from (**I**). WT: wild-type. Met1-sl: methionine 1 start-loss variant. CTCF: corrected total cell fluorescence. Scale bars: 20 μm. ****P ≤ 0.0001, ***P ≤ 0.001, **P ≤ 0.01, *P ≤ 0.05.

The first identified individual was a girl with prenatal ultrasound signs of intrauterine growth retardation (IUGR), skeletal dysplasia, and asymmetric heart cavities. Born at 41 weeks, her weight and length were below the third percentile, with a normal occipitofrontal circumference (31st percentile). During early childhood, she showed global developmental delay (DD), with independent walking achieved at 3 years old and first spoken words at 4. She required special education by age 6 and exhibited severe aggressive behavior and anxiety. Neuropsychological assessment confirmed intellectual disability (ID) (Supplemental Table 1; supplemental material available online with this article; https://doi.org/10.1172/JCI182100DS1)). At 6 years old, she presented with several facial dysmorphic features, such as a long and oval-shaped face, broad forehead, thin lips, low-set ears, depressed nasal bridge and anteverted nares (Individual 1 [I1] — Figure 1A). She had wrinkled skin, suggestive of cutis laxa, and exhibited hyperpigmentation. She had short limbs, hands, and fingers, and limb X-rays revealed advanced carpal and tarsal ossification (Figure 1B). Additional features included umbilical hernia, microphthalmia, ventricular hypertrophy, pulmonary artery atresia, and cerebellar dysplasia. By thirteen, she started developing perceptive hearing impairment. Array-CGH and clinical trio exome sequencing (affected individual and parents) failed to detect pathogenic variants in OMIM-morbid genes. Research reanalysis identified a rare de novo heterozygous start-loss variant in *PTBP1* (NM_002819.5:c.2T>C, p.Met1?) (Figure 2A — supplemental information on variant-filtering criteria).

Through data sharing with the national French Genomic Initiative (PFMG2025-laboratory AURAGEN) or with international laboratories using the GeneMatcher (53) platform, 23 other individuals with *PTBP1* start-loss variants were identified (Figure 2 and Supplemental Tables 1 and 2), all displaying overlapping phenotypes with variable associations of skeletal dysplasia, short-limbed short stature, and DD (Figure 1A). Three other individuals were subsequently identified with missense variants located within the NLS or NES/NLS signals at the N-terminal part of the protein: NM_002819.5:c.41G>A (p.Arg14Gln), NM_002819.5:c.137A>C (p.Lys46Thr), and NM_002819.5:c.144A>T (p.Lys48Asn), (Figure 2A and Supplemental Tables 1 and 2). Interestingly, their phenotype completely overlapped with that of individuals having start-loss variants, except for cleft palate in those with p.Lys46Thr and p.Lys48Asn (Supplemental Table 1). All variants were absent from the gnomAD database (v.4.1.0) (54). Most of them occurred de novo (23/27); in 2 siblings, the variant was inherited from their symptomatic mother, and segregation analysis was unavailable for 2 other individuals.

Overall, the cohort was composed of 15 females and 12 males. One (Individual 24) was born to consanguineous parents. Prenatal ultrasound was abnormal in 13 (48%), revealing short femora (5 of 13, 38%), IUGR (31%), hydramnios (2 of 13, 15%), increased nuchal translucency (15%), asymmetry of heart cavities (1 of 13, 8%), and bilateral hydronephrosis (8%). It led to the diagnosis of skeletal dysplasia in 2. Nineteen were born at term (70%), 7 preterm (26%), and one lacked data. Four individuals (15%) had birth length beyond 2 SDs below the mean, and 3 (11%) had birth weight below –2 SD.

Skeletal anomalies were one of the most striking clinical features and were reported in 24 individuals (89%) (Figure 1B). A variable combination of short stature, short limbs, and skeletal anomalies was observed. Disproportionately short stature and limbs were noted in 17 individuals (63%). A variable combination of short stature and skeletal anomalies was observed. Radiographic features included brachymetacarpia, brachymetatarsia, brachydactyly, brachytelephalangy, brachymesophalangy, and rhizomelia, alongside overlapping toes, syndactyly, and joint hyperlaxity. Advanced bone maturation, cone-shaped epiphyses, and other features such as vertebral dysplasia were also observed (Figure 1B and Supplemental Table 1).

Facial dysmorphism was present in 25 individuals (93%), with shared features including long and oval-shaped face, broad forehead, thin and sparse eyebrows, epicanthal folds, depressed nasal bridge and thin upper lip (Figure 1A). We generated a computer-assisted facial composite using the Facer toolkit (supplemental information), which confirmed the most prominent morphological features of the PTBP1-related disorder (Figure 1A).

DD was noted in 78%, behavioral problems in 30%, and ID in 26% of individuals, generally mild to moderate (Supplemental Table 1). All individuals were ambulatory on examination. Behavioral problems included autism spectrum disorder (ASD), anxiety, aggression, and attention disorder, with or without hyperactivity. One (I 15) had psychogenic nonepileptic seizures.

Neurological examinations were mostly unremarkable or limited to signs related to DD (70%). Three individuals (11%) displayed upper-motor–neuron signs, which were mostly mild and limited to brisk tendon reflexes and ankle clonus. Three (11%) exhibited axial hypotonia, 1 (4%) presented with (limb) dystonia, and another with cerebellar ataxia.

Brain MRI in 18 individuals revealed abnormalities in 43% (8 of 18), including cerebellar hypoplasia/dysplasia (2 of 8), corpus callosum hypoplasia (1 of 8), ventriculomegaly (2 of 8), white matter T2-weighted signal hyperintensities compatible with hypomyelination (1 of 8), double and ectopic pituitary gland (1 of 8), mega cisterna magna (1 of 8), and septo-optic dysplasia (1 of 8). One individual (I 4) was diagnosed with multiple sclerosis at age 27 and presented with congruent white matter anomalies.

Additional clinical features included skin, nail, and hair anomalies (52%), dental anomalies (37%), abnormal ophthalmological findings (44%), and cardiovascular defects (22%) (Supplemental Table 1). Metabolic problems (22%) included hard-to-control hypoglycemia (*n* = 2), low IGF-1 (*n* = 2) (with one individual having normal levels of growth hormone), and elevated total and LDL cholesterol (*n* = 1).

### PTBP1 start-loss variants result in alternate translation initiation.

*PTBP1* is highly intolerant to loss-of-function (LoF) variants, with an associated probability of LoF intolerance (pLI) score of 1.0 (gnomAD v4.1.0), LoF observed/expected upper bound fraction (LOEUF) score of 0.25, and a predicted probability of haploinsufficiency (pHaplo) score of 0.97 by DECIPHER (55). The missense and synonymous variation constraint of *PTBP1* is relatively low (Z-score = 1.076). To investigate the impact of heterozygous start-loss and missense variants on PTBP1 expression, we performed Western blot analyses on protein extracts from primary fibroblasts of 4 individuals with c.2T → C (I 1 and I 16), c.1A → G (I 2), and c.3G → A (I 4) variants, and from healthy (control) individuals (Figure 2B). In control samples, a major band corresponding to the comigrating isoforms PTBP1-4 and PTBP1-2 (557 and 550 AAs, respectively; approximately 57 kDa) was observed, along with a fainter band corresponding to PTBP1-1 (531 AAs), in line with previous observations (50, 56). In contrast, samples from affected individuals showed altered isoform ratios, with a reduced upper band and increased intensity of the lower one (a and a*+b, respectively; Figure 2B), alongside an additional faint and lower-migrating band (b*; Figure 2B), suggesting alternate translation from an in-frame start codon approximately 25–30 AAs downstream. This pattern was associated with an overall 1.4-fold increase in PTBP1 expression (Figure 2C). To investigate this, we performed site-directed mutagenesis in plasmids encoding either PTBP1-4 or PTBP1-1 isoforms in-frame with a C-terminal turbo-GFP (i.e., PTBP1-4/PTBP1-1-tGFP) (Supplemental Figure 1, A and B) to model human start-loss and missense variants (i.e., p.Arg14Gln, p.Lys46Thr, and p.Lys48Asn). We also modeled the NM_002819.5:c.137A → G (p.Lys46Arg) variant that had been reported in gnomAD with tolerated predictions of pathogenicity and identified in an individual with African/African American ancestry as a benign missense variant. Here, lysine is replaced with an arginine, presenting with the same biochemical properties (basic and positively charged). Each construct was transiently expressed in transfected NIH-3T3 cells, and Western blot analysis documented the expected migration profile for WT PTBP1 and the benign p.Lys46Arg variant (approximately 80 kDa) (Supplemental Figure 1, C and D). In contrast, the start-loss constructs resulted in shorter chimeric proteins in line with the findings collected in primary fibroblasts from start-loss individuals (Supplemental Figure 1, C and D). These results were independently validated using anti-tGFP and anti-PTBP1 antibodies. Consistent with these findings, in silico analyses showed the presence of a second in-frame methionine located 30 AAs downstream of the canonical start codon (Supplemental Figure 2A), suggesting that start-loss variants result in shorter proteins lacking the first 30 AAs of the N-terminal region encompassing the first half of the bipartite NLS element overlapping with the NES (Figure 2A and Supplemental Figure 2B). The in silico Kozak consensus sequence prediction tool ATGpr (57) pinpointed Met31 in PTBP1 (reliability 0.63) as a candidate downstream alternative start codon (Supplemental Figure 2C). To test that hypothesis, we performed site-directed mutagenesis of this codon (c.92T → C) in PTBP1-4-tGFP already coding the c.2T → C start-loss variant. We observed that, without PTBP1-Met31 (Supplemental Figure 2D), the product as detected by the anti-tGFP antibody was even shorter, confirming that Met31 is indeed used as an alternate start codon.

Finally, to further investigate the increase in PTBP1 expression observed in immunoblot, we performed a cycloheximide (a eukaryotic translational elongation blocker) chase assay. It revealed increased stability of the mutated protein, suggesting a potential regulatory role of N-terminal residues in WT protein degradation and turn over (Figure 2, D and E).

### Disease-associated start-loss and missense variants affecting the NES/NLS disrupt the steady-state nucleocytoplasmic distribution of PTBP1.

The N-terminal region of PTBP1 is highly conserved across species (Supplemental Figure 2B). The location of the identified variants suggested a possible disturbance of the nucleocytoplasmic distribution of the mutated proteins. We therefore performed direct PTBP1 immunostaining coupled with actin and Hoechst counterstain analysis in primary fibroblast cell lines derived from controls or individuals with start-loss variants, allowing us to precisely quantify PTBP1 expression in cytoplasmic and nuclear compartments (Figure 2, F and G).

Although fibroblasts from affected individuals showed no differences in nuclear or cytoplasmic size compared with controls (Supplemental Figure 3A), start-loss cell lines exhibited increased cytoplasmic distribution and expression of PTBP1 accompanied by reduced nuclear localization (Figure 2, F and G). This suggests that the increased availability of PTBP1 is unevenly distributed between cellular compartments. Quantitative and qualitative differences in PTBP1 distribution were also corroborated by Western blot analysis on fractionated subcellular components, which showed a specific increased stability and retention of the mutated protein isoforms in the cytoplasm, but not in the nucleus, ruling out a possible dominant-negative effect of the mutated proteins (Supplemental Figure 3, B and C).

To overcome the confounding signal generated by the WT allele and to model the missense variants’ effect, we also analyzed the distribution of the chimeric tGFP-tagged proteins in transiently transfected NIH-3T3 mouse fibroblast cells (Figure 2H). This cell line shows a higher nucleus-cytoplasmic ratio (N/C ratio= 1/2) than human fibroblasts (N/C ratio= 1/5), allowing us to better appreciate the potential altered nucleocytoplasmic distribution associated with the tested variants. Quantitative analysis estimated WT PTBP1 to be approximately 80% nuclear and approximately 20% cytoplasmic at steady state (Figure 2I). In contrast, start-loss variants showed a weaker signal in the nuclear compartment, corresponding to an approximately 30%–35% decrease, accompanied by an opposite increase in the cytoplasmic compartment compared with WT, an observation in keeping with the imbalanced distribution observed in primary fibroblasts from PTBP1 start-loss individuals. Missense variants showed a similar but greater effect on PTBP1 localization, estimated at approximately 35%–40%, associated with p.Lys46Thr and p.Lys48Asn variants, affecting only the second half of the bipartite NLS (Figure 2, I and J). Intriguingly, the effect of the p.Arg14Gln variant, located within the first half of the bipartite NLS overlapping the NES, was very similar to what was observed in start-loss transfected cells.

Human variants induced the same outcome when transfected in NIH-3T3 cells using the PTBP1-1 isoform (Supplemental Figure 3D). Because of the overlapping phenotypes induced by mutated PTBP1-4 and PTBP1-1 isoforms, we decided to pursue the rest of the study using only PTBP1-4.

### Pathogenic variants in PTBP1 are associated with a specific DNA methylation episignature.

**Figure 3:**
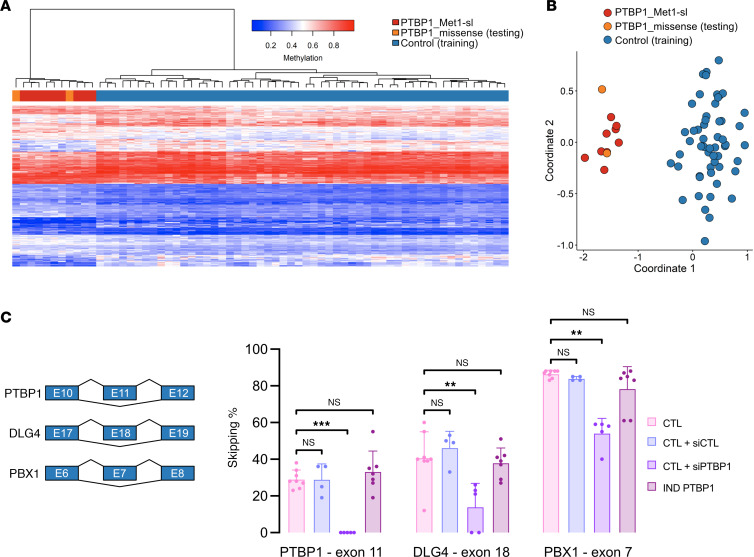
Molecular characterization of pathogenic PTBP1 variants. (**A**) Heatmap and (**B**) multidimensional scaling (MDS) plot showing PTBP1 episignature clustering of start-loss (red), matched controls (blue), and missense cases (orange). Columns, PTBP1 individuals or controls; rows, selected probes. (**C**) Targeted cDNA amplicon sequencing of exon-skipping events in PTBP1 (exon 11), DLG4 (exon 18), and PBX1 (exon 7) transcripts from start-loss fibroblasts, untreated controls, and controls treated with either scramble (negative control) or PTBP1-specific siRNA (positive control). Mann–Whitney U test. *n* = 4 independent experiments.

The development of DNA methylation episignatures as effective biomarkers to assess the pathogenicity of variants associated with specific rare diseases is an emerging field of interest in human molecular genetics (58–60). DNA methylation analysis of peripheral blood–derived samples, using Illumina Infinium MethylationEPIC v1 BeadChip arrays, from 9 individuals carrying PTBP1 start-loss variants allowed us to identify differentially methylated probes and regions that were used to train a support vector machine–based (SVM-based) model (episignature) as a specific biomarker classifier. We observed separate grouping of cases and controls in both heatmap and multidimensional scaling plots (Supplemental Figures 4, 5, and 6, and supplemental information). We used the developed model to investigate the episignature of 2 individuals with missense variants in PTBP1. In keeping with the localization results obtained in the transfection assays, DNA samples from patients with missense variants also exhibited an episignature compatible with start-loss samples (Figure 3, A and B), indicating the presence of converging molecular mechanisms between the 2 variant classes.

### PTBP1 start-loss variants are not associated with splicing defects in primary fibroblasts and colocalize to processing bodies.

Our results showed a dual effect of pathogenic variants in PTBP1, namely reduced nuclear localization and enhanced cytoplasmic retention and stability.

To assess whether the remaining nuclear fraction of PTBP1 maintained its major role as a splicing regulator, we used targeted cDNA amplicon sequencing to detect possible aberrant splicing events in primary fibroblast cell lines obtained from individuals with PTBP1 start-loss variants. As a positive control of our experiment, we also carried out PTBP1 knockdown using RNA interference, obtaining a residual expression below 20% (Supplemental Figure 7A). We then performed splicing analysis on well-established targets namely exon 11 in PTBP1, exon 18 in DLG4, and exon 7 in PBX1, as a readout of PTBP1 exon-skipping activity (30, 32, 56). For all 3 RNAs, the exclusion rates observed in control cells were comparable with those observed in cells treated with scrambled siRNA controls. Conversely, we saw significant deregulation in si*PTBP1*-treated cells (Figure 3C). Interestingly, cells with PTBP1 start-loss variants showed similar levels of exon skipping to control cells. These results were corroborated by global mRNA-seq, where PTBP1 down-regulation (si*PTBP1*-treated cells) led to a significantly higher number of aberrant splicing events compared with fibroblast lines treated with scramble siRNAs or untreated control cell lines (Supplemental Figure 7B). In particular, splicing anomalies included alternative 5′ and 3′ splice sites, skipped exons, and mutually exclusive exons (Supplemental Figure 7C). Altogether, these data indicate that the nuclear decrease of PTBP1 associated with heterozygous start-loss variants affecting the NES/NLS does not alter its global splicing activity.

**Figure 4:**
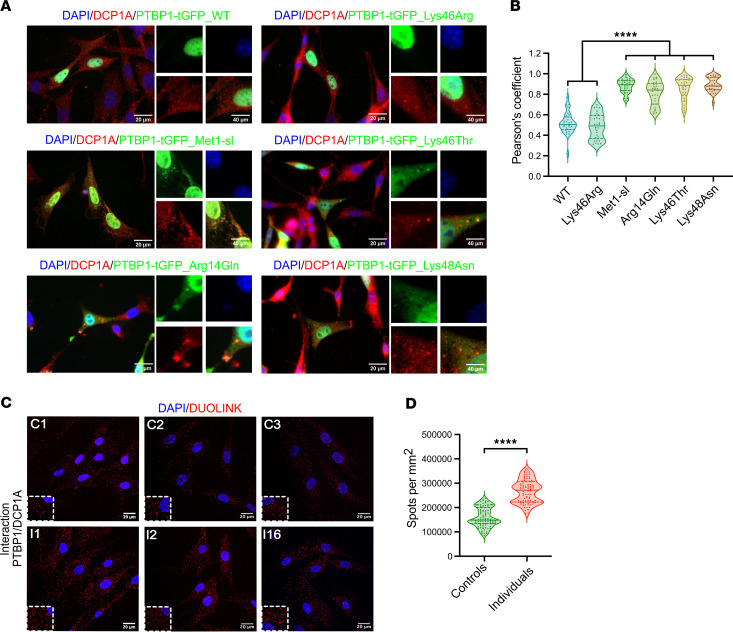
Cytoplasmic colocalization of start-loss PTBP1 variants with P-bodies. (**A**) Imaging of NIH-3T3 cells transfected with WT, start-loss or missense PTBP1-4-tGFP constructs (green), immunostained for DCP1A (red); DAPI (blue). *n* = 5 independent experiments. (**B**) Pearson’s correlation of DCP1A/PTBP1 colocalization. *n* = 30 cells/condition; Mann–Whitney U test. (**C**) Proximity ligation assay (PLA) of PTBP1–DCP1A interactions in PTBP1 start-loss fibroblasts and controls. *n* = 3 independent experiments. (**D**) Quantification of PLA puncta shown in **C**. *n* = 90 cells per condition; Student’s *t* test. Met1-sl, methionine 1 start-loss variant. Scale bars: 20 μm. ****P ≤ 0.0001, ***P ≤ 0.001, **P ≤ 0.01, *P ≤ 0.05.

Next, we sought to refine the cytoplasmic localization of PTBP1. This protein is involved in stress granule assembly during infection by transmissible gastroenteritis virus (TGEV) coronaviruses and mouse hepatitis coronavirus (MHC) (61). We therefore hypothesized that its subcellular localization could be linked to cytoplasmic structures related to RNA metabolism and gene expression. We performed coimmunostaining for PTBP1 and G3BP stress granule assembly factor 1 (G3BP1) (MIM 608431), which is implicated in stress granule nucleation, and decapping mRNA 1A (DCP1A) (MIM 607010), an enzyme that is part of an RNA degrading complex associated with NMD in processing bodies (P-bodies) (62, 63). We also used targets unrelated to RNA metabolism as negative controls, including early endosome antigen 1 (EEA1) (MIM 605070), which is involved in endosome trafficking, and the Golgi-associated protein GOPC/PIST (MIM 606845) (64, 65). Confocal imaging of NIH-3T3 cells transfected with the PTBP1-4-tGFP start-loss construct revealed no colocalization with the 3 markers G3BP1, EEA1, and GOPC/PIST (Supplemental Figure 8A). However, PTBP1-4-tGFP containing pathogenic start-loss and missense variants showed colocalization with DCP1A, with a Pearson’s coefficient calculated for the above PTBP1-4 mutants close to 0.9 (Figure 4, A and B), significantly higher than in cells transfected with the WT or p.Lys46Arg plasmid or compared with the markers G3BP1, EEA1, and GOPC/PIST (below 0.5, Supplemental Figure 8B).

To test whether PTBP1 and DCP1A were in close vicinity, we performed a proximity ligation assay in primary fibroblasts derived from start-loss and healthy (control) individuals. This technique allows the detection of protein–protein interactions in situ at distances under 40 nm. As PTBP1 and G3BP1 do not colocalize in normal conditions, we estimated their proximity as a reference background for our experiment (Supplemental Figure 8C). As expected, no signal was detected in either control or PTBP1 start-loss fibroblast cell lines. However, we did observe positive foci between PTBP1 and DCP1A in control cells (Figure 4, C and D) indicating that PTBP1 interacts with DCP1A, and more generally with processing bodies, under physiological conditions. In contrast, the number of positive signals in start-loss cells was far higher (Figure 4, C and D), although the total of DCP1A-positive foci did not significantly change in start-loss cells compared with control lines (Supplemental Figure 8, D and E), indicating that pathogenic PTBP1 variants increased the likelihood (by 1.6×) of interaction with processing bodies without altering their abundance.

### PTBP1 regulates the stability of mRNAs involved in osteochondrogenic and neurodevelopmental pathways.

In addition to splicing, PTBP1 is involved in many other biological aspects of RNA biogenesis, including transport, localization, stability, and translation. We observed that variants causing cytoplasmic retention and accumulation increased the likelihood of PTBP1 to colocalize with P-bodies, which are cytoplasmic ribonucleoprotein granules primarily composed of translationally repressed mRNAs and proteins related to 5’-to-3’ mRNA decay, and therefore implicated in posttranscriptional regulation. We hypothesized that at least part of the RNAs associated with PTBP1 would show altered stability (63).

**Figure 5:**
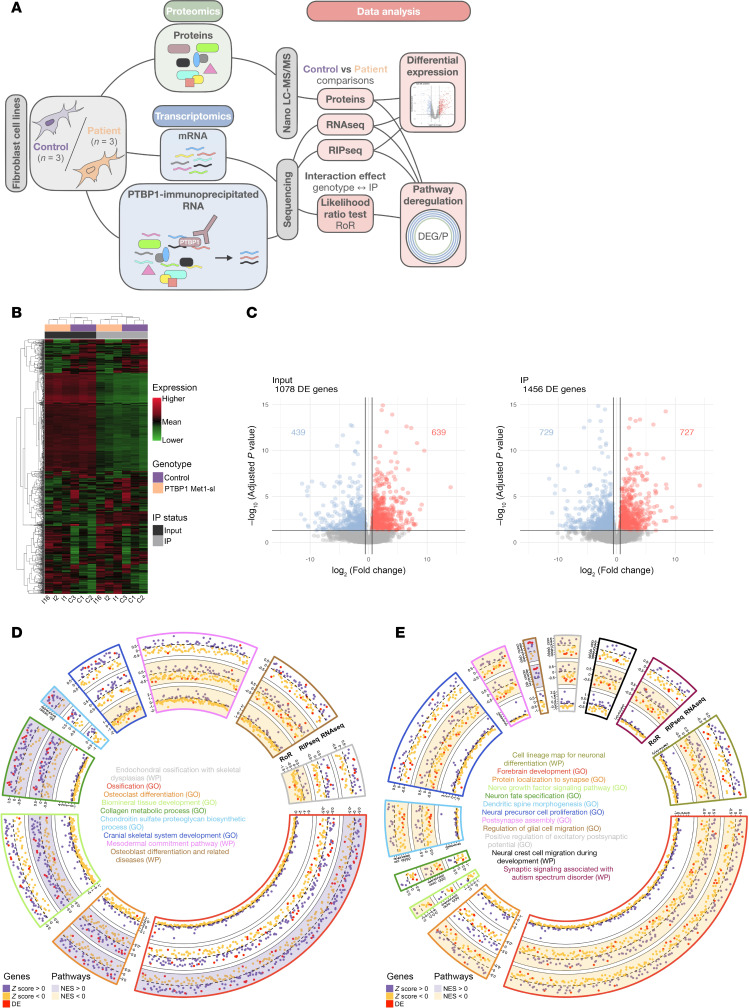
Integrated analysis of patient-derived fibroblasts with start-loss PTBP1 variants. (**A**) Workflow for RNA-seq, RIP-seq, and proteomic analyses in fibroblasts from controls and PTBP1 start-loss individuals. RoR = IP/input ratio in start-loss versus controls. (**B**) Hierarchical clustering of the top 5% most variable genes (*n* = 1,184) in input or IP fractions. (**C**) Volcano plots of differential gene expression in input (top) and PTBP1-IP (bottom) fractions. (**D** and **E**) Circular plots of deregulated mesodermal (**D**) and neurodevelopmental (**E**) pathways based on RNAseq (outer), RIP-seq (middle), and RoR (inner) Z-scores.

To explore the biological consequences of PTBP1-altered nucleocytoplasmic distribution at a transcriptional level, we also carried out RNA immunoprecipitation sequencing (RIP-seq) with either an antibody against PTBP1 (PTBP1-IP) or an IgG (mock-IP) from previously extracted RNA, which served as input material (input RNA-seq) for our experiment on PTBP1 start-loss and control fibroblast lines (Figure 5A). Library enrichment of PTBP1-associated RNAs (PTBP1-IP) was calculated by comparing gene expression over the input and the mock-IP, revealing the presence of transcripts specifically associated with PTBP1 (Supplemental Figure 9A). Principal component analysis (PCA) on raw RIP-seq data revealed that the first 2 components accounted for 68% of the total variance. The first principal component (PC1, 41.7%) primarily separated input and IP samples, while the second (PC2, 20.7%) distinguished control from start-loss samples (Supplemental Figure 9B). Hierarchical clustering of the 5% most variable detected genes expressed across all conditions revealed the presence of distinct patterns of coregulated genes according to input/IP conditions and genotype (Figure 5B). Next, we conducted a differential expression analysis to compare control and PTBP1 start-loss samples across input and IP conditions identifying 1,078 and 1,456 differentially expressed (DE) genes, respectively, corresponding to a deregulation of 5.5% and 7.4% of the expressed genes detected in the dataset (19,572 genes) (Figure 5C). The analysis also revealed that 872 were shared by the 2 conditions (Supplemental Figure 9C) corresponding to 80.9% and 59.9% of DE genes, respectively.

Next, we investigated dysregulated biological pathways between control and start-loss groups emerging from the transcriptomic data using gene-set enrichment analysis (GSEA). All detected genes were ranked according to their fold change and adjusted *P* value for input and IP RNA sequencing experiments. Using WikiPathways (WP) and Gene Ontology (GO) databases for annotation (66–68), GSEA identified 488 and 441 deregulated biological pathways in the input RNA-seq and the RIP-seq datasets, respectively, of which 304 were in common. To overcome potential bias due to deregulated genes in control versus PTBP1 start-loss conditions, we employed a likelihood-ratio test (lrt) to analyze the differential enrichment between immunoprecipitated fractions over the basal expression observed in input samples (RoR: ratio of ratios) (69). This analysis allowed us to identify 186 DE genes, 136 of which (73.1%) were not shared with either the input or IP groups, as well as 186 unique additional pathways that differed between control and start-loss fibroblasts (Supplemental Figure 9C and Supplemental Tables 3 and 6). Interestingly, among those, 36 were related to clinical anomalies observed in our cohort with 12 linked to mesodermal commitment/skeletal development and 24 to neurodevelopment. After removing redundant terms, we identified 9 and 12 unique pathways associated with 98 and 92 differentially expressed genes, identified in at least one of the 3 tests (i.e., input, RIP-seq, lrt), respectively (Figure 5, D and E).

**Figure 6:**
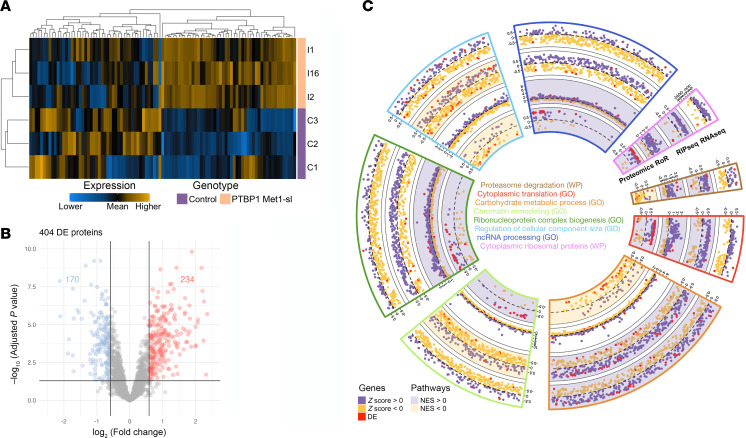
Proteomic dysregulation in fibroblasts from individuals with start-loss PTBP1 variants and associated biological pathways. (**A**) Volcano plot of differential protein expression relative to controls. (**B**) Hierarchical clustering of the top 5% most variable proteins (*n* = 97). (**C**) Circular plot of pathways commonly deregulated at RNA and protein levels. From outer to inner: RNA-seq, RIP-seq, and RoR Z scores, and proteomics label-free quantification intensities. Red dots, significantly deregulated genes/proteins. Background colors indicate the normalized enrichment score (NES) direction from Gene/Protein Set Enrichment Analysis (G/PSEA).

Finally, to explore the impact of the increased likelihood of PTBP1 colocalization with processing bodies on protein synthesis, we performed a quantitative proteomic analysis using nano-liquid chromatography followed by tandem mass spectrometry (Nano LC-MS/MS) on 6 protein samples extracted from 3 PTBP1 start-loss and 3 control fibroblast cell lines (Figure 5A). Hierarchical clustering of the 5% most variably detected protein expressions across the 2 conditions revealed the presence of distinct patterns of coregulated proteins (Figure 6A). It identified 1,942 protein isoforms (i.e., 9.9% of the 19,572 genes considered in the transcriptomic analysis), of which 404 were differentially expressed with an absolute fold change greater than 1.5 (Figure 6B and Supplemental Table 3). Among these, 48 overlapped with genes identified in the transcriptomic analysis (Supplemental Figure 9C). Following the ranking method previously described, we identified 100 deregulated biological pathways, encompassing a broad range of biological processes (67, 68, 70), with a fifth of them in common with those identified in our transcriptomic analysis. After removing redundant terms, we identified 8 unique terms with either a concordant (5 of 8) or discordant (3 of 8) NES between transcriptomics and proteomic data. In particular, the chromatin remodeling pathway was downregulated in the IP transcriptomic condition while it was upregulated in proteomic data. In contrast, carbohydrate metabolic process and proteasome degradation were upregulated in the transcriptomic data (input-IP and IP-lrt respectively), but downregulated in the proteomic data. Even though our proteomic analysis could not extend beyond 10% of the corresponding transcripts detected by transcriptomic analysis, these results support the idea that processing bodies add a layer of regulation. By participating in RNA storage and degradation, they may play a pivotal role at the interface between transcriptomic and proteomic levels, potentially leading to opposite expression between RNA and proteins. Interestingly, some of them suggested an involvement of the altered nucleocytoplasmic distribution of PTBP1 in protein biogenesis, including translation and degradation processes (Figure 6C). A total of 169 unique genes/proteins were identified as differentially expressed in at least 1 analysis (Supplemental Table 3).

### Human start-loss and missense variants affecting NLS/NES functions show altered nucleocytoplasmic distribution in vivo.

**Figure 7:**
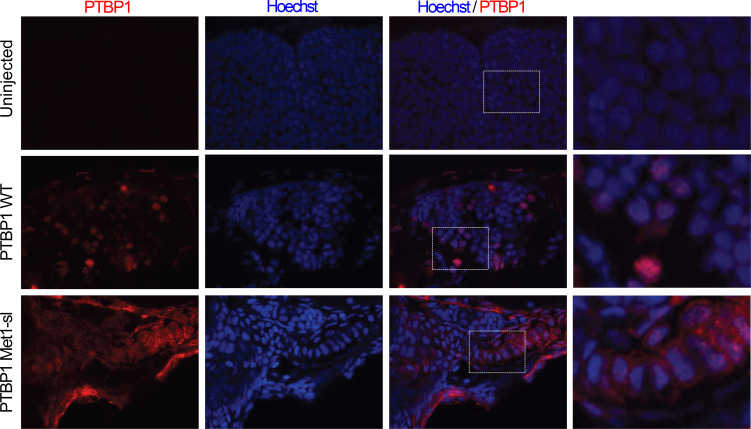
Expression of human PTBP1 variants in zebrafish. PTBP1 (red) immunofluorescence in 5 dpf zebrafish embryos injected at the one-cell stage with human wild-type (nuclear localization) or start-loss (cytoplasmic retention) mRNA. Hoechst (blue). Endogenous zebrafish ptbp1 is not detected. Representative images show samples under different magnifications. Original magnification, ×20 (columns I, II, and III); ×60 (column IV). In column IV, views of the areas outlined by dotted rectangles in column III are shown, highlighting finer structural details within the selected regions. *n* = 3 independent experiments.

To assess the differential localization and biological activity of human variants in vivo, we took advantage of Danio rerio, a gold standard vertebrate model for neurodevelopmental and skeletal disorders. PTBP1 expression is conserved among vertebrates, and during zebrafish embryogenesis its orthologous mRNAs (ptbp1a and ptbp1b) are expressed in the developing brain, branchial arches, and fin primordia — structures that give rise to craniofacial and pectoral fin cartilages, respectively — among others (Supplemental Figure 10A). According to Decipher, *PTBP1* has a predicted triplosensitivity score (pTriplo) of 0.66, suggesting a potential dosage-sensitive mechanism in humans (pTriplo ≥ 0.68 indicate an odds ratio ≥ 2). Hence, we hypothesized that Danio rerio development could serve as a readout for the in vivo effects of exogenous human PTBP1 expression. Zebrafish embryos were injected with either in vitro transcribed WT or start-loss human PTBP1 mRNAs at the 1-cell stage. Immunofluorescence analysis in uninjected embryos revealed that anti-human PTBP1 did not cross react with endogenous proteins. WT human PTBP1 was mainly distributed in the nucleus in injected embryos, while the start-loss PTBP1 was also expressed in the cytoplasm at 120 hpf (Figure 7), recapitulating the cytoplasmic retention observed in vitro in fibroblasts derived from affected individuals and in transfected NIH-3T3 cells.

**Figure 8:**
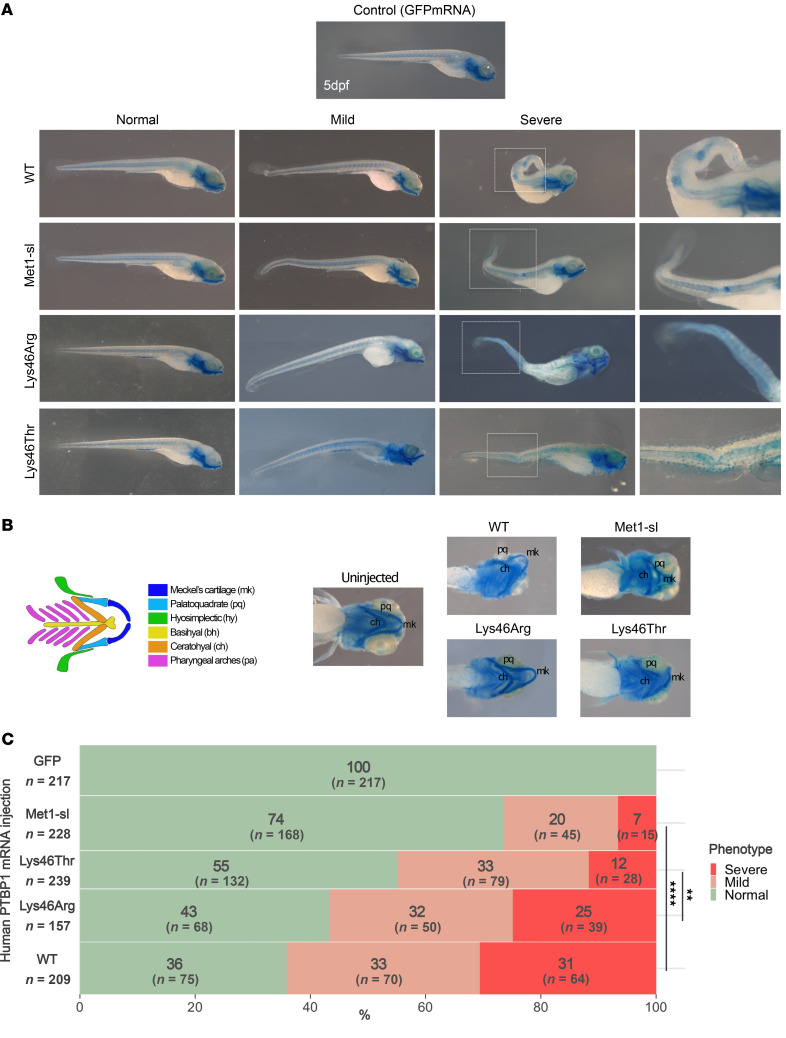
Phenotypic analysis of zebrafish embryos injected with human PTBP1 mRNA at the one-cell stage. (**A**) Lateral views of Alcian Blue-stained embryos at 5 dpf reveal caudal fin defects in WT and start-loss PTBP1-injected animals. The mild phenotype observed in embryos injected with Lys46Arg and Lys46Thr PTBP1 consist of slight or mild spine curvatures; severe cases show trunk and caudal fin deformities and ectopic staining (boxed). (**B**) Ventral view at 120 hpf showing craniofacial cartilage defects in severely affected embryos. (**C**) Stacked bar plot showing the phenotypic distribution of mild/severe defects across embryos injected with WT, benign (Lys46Arg), or pathogenic variants (start-loss and Lys46Thr). The number of the injected embryos per condition is indicated in the figure. χ^2^ test. Met1-sl, methionine 1 start-loss variant. Dpf, days post fertilization. ****P ≤ 0.0001, ***P ≤ 0.001, **P ≤ 0.01, *P ≤ 0.05.

Since skeletal dysplasia was a recurrent feature in our patient cohort, we employed Alcian Blue skeletal staining in zebrafish as a functional readout to assess the differential activity of the human variants. At 120 hpf, organogenesis is complete, and embryonic cartilages are differentiated. The phenotypes of injected embryos were classified based on their severity into 3 categories: normal, mild, and severe. Normal larvae were indistinguishable from those injected with GFP mRNA alone (Figure 8A). Larvae with mild phenotypes showed malformations of the spine and caudal fin, whereas larvae with severe phenotypes showed curvature of the spine with vertebral dysplasia in the caudal region, and the formation of ectopic cartilage foci. These embryos also showed craniofacial abnormalities involving Meckel’s cartilage, corresponding to the future zebrafish jaw (Figure 8, A and B). Embryos injected with WT PTBP1 resulted in two thirds of larvae with mild (1 of 3) or severe (1 of 3) phenotypes. The same result was obtained with the transcript carrying the benign variant p.Lys46Arg. However, those injected with either the pathogenic p.Lys46Thr or start-loss variants resulted in a dramatic reduction of affected embryos, with larvae presenting with severe phenotypes plummeting to almost 12% and 6% (Figure 8C). Overall, these results confirmed that pathogenic variants altering nucleocytoplasmic distribution show an altered biological activity as compared with WT- and p.Lys46Arg-expressing embryos in vivo.

### Individuals with PTBP2 variants altering nucleocytoplasmic localization show nonsyndromic neurodevelopmental and behavioral disorders.

PTBP1 and PTBP2 share over 75% protein sequence homology, particularly at the N-terminal region containing the NES and NLS sequences. Data sharing led to the identification of 2 unrelated male individuals with de novo start-loss NM_021190.4:c.2T>C (p.Met1?) or missense NM_021190.4:c.41G>C (p.Arg14Thr) variants in PTBP2 (Supplemental Figure 11A and Supplemental Table 2). The latter had been reported in a series of individuals with severe childhood speech disorder (71). Both were born after uneventful pregnancies with normal prenatal ultrasounds and normal birth parameters. They both presented with DD, ID, and behavioral problems, which included autistic features and attention-deficit/hyperactivity disorder. However, their phenotype did not expand much beyond the scope of neurological impairment as neither presented with skeletal anomalies (only mild brachydactyly for I 29), skin, nail, hair or teeth anomalies, or other evident clinical features such as heart defects. Abnormal facial features were observed in Individual I 28, which were congruent with those observed in PTBP1 individuals (oval face, high forehead, thick eyebrows, and synophrys). Neurological examinations revealed orofacial motor tics in I 28, who also presented with generalized tonic-clonic seizures. Brain MRIs were normal in both cases (Supplemental Table 1).

PTBP2 is constrained for missense (Z score = 3.45 according to gnomAD v.4.1.0) and loss-of-function variants (pLI = 1; LOEUF = 0.42). We performed site-directed mutagenesis to obtain C-terminal tGFP-tagged PTBP2 (PTBP2-tGFP) proteins containing either the human start-loss or p.Arg14Thr variants. Upon transient transfection in NIH-3T3 cells, protein expression was assessed by Western blot (Supplemental Figure 11C). The start-loss variant induced a protein shift comparable with what was observed for PTBP1 start-loss variants. In silico Kozak consensus sequence prediction analysis suggested Met32 and Met53 in PTBP2 (reliability 0.15 and 0.21 respectively) as candidate downstream alternate start codons (Supplemental Figure 2, A–C). To test this hypothesis, we performed site-directed mutagenesis of these codons in PTBP2-tGFP already harboring the start-loss variant. We observed that PTBP2-Met32Thr or -Met53Thr (Supplemental Figure 3D) are dispensable for translation initiation, although they appeared to reduce protein synthesis slightly. We also performed site-directed mutagenesis on another nearby methionine, PTBP2-Met35Thr, in the context of start-loss alone or in combination with mutagenized Met32 (Supplemental Figure 11D) obtaining a reduction in PTBP2 translation only when Met1, Met32, and Met35 were all mutated. Similar to PTBP1, NIH-3T3 cells transfected with PTBP2-tGFP plasmid carrying start-loss or p.Arg14Thr variants demonstrated cytoplasmic retention (Supplemental Figure 12, A and B) and colocalization with processing bodies (Supplemental Figure 12C). Pearson’s coefficient was 0.8 for DCP1A colocalization of PTBP2-tGFP encoding start-loss or p.Arg14Thr variants while it was below 0.5 for the WT PTBP2-tGFP protein (Supplemental Figure 12D).

Finally, genome-wide transcriptome analysis from primary fibroblasts obtained from the individual with the de novo p.Arg14Thr variant in PTBP2 showed no major splicing defects (Supplemental Figure 7B) and a transcriptional signature comparable with samples obtained from PTBP1 in PCA analysis (Supplemental Figure 9B) and hierarchical clustering (Supplemental Figure 13) overall, suggesting that variants affecting nucleocytoplasmic distribution in PTBP1 and PTBP2 may share common pathophysiological mechanisms.

## Discussion

We report on a collaborative study of 27 individuals with pathogenic variants in PTBP1, all affecting the protein’s nucleocytoplasmic distribution. These variants have a dual effect: reduced nuclear localization and enhanced cytoplasmic retention, with start-loss variants also leading to increased protein stability. This highlights the importance of tightly regulated subcellular localization of PTBP1 function during development.

Clinically, the phenotype has 3 key components. The most striking is skeletal dysplasia, which is both specific and consistent across affected individuals. It features advanced bone maturation, short and dysplastic bones, and disproportionate body measurements. Transcriptomic analyses of patient-derived fibroblasts show the deregulation of pathways linked to mesoderm commitment, osteoblast/osteoclast differentiation, ossification, and collagen metabolism.

The second major feature is facial dysmorphism. Cleft palate was specifically associated with the p.Lys46Thr and p.Lys48Asn variants, but not in those with start-loss or p.Arg14Gln variants. These mutations affect only the second stretch of the NLS, resulting in greater cytoplasmic retention and P-body colocalization.

The third aspect is neurodevelopmental involvement, present in 78% of individuals, with variable severity from isolated fine motor delays to ID (in 26%). When present, ID ranged from mild to moderate. Given the limited number of non-start-loss variants, genotype/phenotype correlations remain speculative at this stage. Interestingly, transcriptomic analyses identified altered pathways involved in neurodevelopment, such as regulation of neural precursor cell proliferation, migration, and synapse formation.

At a molecular level, our studies demonstrate that nucleocytoplasmic distribution is central to PTBP1-related disease. Start-loss variants lead to translation from the second in-frame methionine, eliminating the first 30 AAs, including the first half of the bipartite NLS that overlaps with the NES. These variants were associated with reduced nuclear localization of PTBP1, accompanied by increased cytoplasmic retention and protein stability, likely contributing to the phenotype. Missense variants substitute basic and positively charged lysine or arginine residues with polar and uncharged threonine, asparagine, or glutamine residues, also affecting nuclear import and causing cytoplasmic retention. Consistently, DNA methylation analysis revealed that start-loss and missense variants have a common episignature, suggesting shared mechanisms between the 2 classes of variants.

Previously, it was demonstrated that the integrity of the bipartite NLS at the N-terminus of PTBP1 is required for translocation by importin α (43), a key mediator of nuclear import (72, 73). Additionally, within the N-terminus, Cys23 is critical for PTBP1 homodimerization in the nucleus. The artificial p.Cys23Ser mutation impairs dimerization and splicing but preserves RNA binding when transfected in HeLa cells (74). Our start-loss variants remove the first 30 AAs, including Cys23, likely preventing dimerization. Western blot analysis on fractionated subcellular components suggests that the mutant protein isoforms, which show increased stability and cytoplasmic retention, do not alter the nucleocytoplasmic distribution of the WT protein. Our targeted and genome-wide RNA-seq experiments document that pre-mRNA splicing is preserved in patient-derived fibroblasts, indicating that the combined nuclear pool of WT and residual mutant PTBP1 may still be sufficient for proper nuclear function. However, alternative explanations cannot be ruled out, including partial functional compensation by PTBP2, in tissues where it is expressed, or subtle cell type–specific effects that could not be evaluated in our study, which relied on fibroblasts. These possibilities warrant further investigation in disease-relevant models.

In vivo, the nucleocytoplasmic distribution and the differential activity of pathogenic variants become more apparent under nonphysiological conditions, such as upon expression of human PTBP1 mRNA in zebrafish embryos injected at the 1-cell stage. This experimental setup allowed us to confirm that pathogenic variants lead to cytoplasmic retention in vivo. However, the model does not permit precise quantification of subcellular localization due to the mosaic distribution of injected mRNA. *PTBP1* shows a predicted score of triplosensitivity close to cut-off, according to Decipher. Notably, embryos injected with predominantly nuclear forms — such as the WT or the benign Lys46Arg variant — developed more severe phenotypes than those expressing variants with cytoplasmic retention (i.e., start-loss or Lys46Thr). This indicates that pathogenic variants exert a differential biological activity compared with control alleles, rather than functioning as simple LOF or neutral variants. Consistent with this observation, we found that PTBP1 normally localizes to processing bodies, but this colocalization is enhanced when start-loss or missense variants result in PTBP1 cytoplasmic retention, suggesting a potential hypermorphic effect with respect to its cytoplasmic function in human fibroblasts. A similar mechanism has been described for missense variants within the localization signal of the RNA-binding protein CELF2 (MIM 602538), which cause the cytoplasmic retention, binding and repression of mRNAs promoting neurogenesis (75). Our data suggest that PTBP1 variants might similarly modulate RNA fate abnormally. Specifically, start-loss variants do not appear to confer novel RNA-binding properties, per se. Rather, they result in increased cytoplasmic retention and enhanced colocalization with P-bodies, which, in turn, secondarily affect mRNA stability and protein expression. Therefore, the observed transcriptomic and proteomic differences are likely a consequence of altered subcellular localization rather than a direct change in RNA-binding specificity. In vivo, the supraphysiological and mosaic expression of human PTBP1 in zebrafish embryos precludes reliable assessment of colocalization with P-bodies, as any observed signal may be artifactual.

Functionally, we found that PTBP1 colocalization with P-bodies correlates with pathways related to protein biogenesis in human fibroblasts. These findings warrant further investigation, ideally through spatial or single-cell transcriptomics in relevant cell types (e.g., induced pluripotent stem cell-derived organoids) and in vivo models (e.g., knock-in mice), to better elucidate the relationship between PTBP1 mislocalization to the cytoplasm and P-bodies, and its role in skeletal dysplasia and neurodevelopmental pathophysiology.

Finally, we identified 2 unrelated individuals with de novo heterozygous variants in PTBP2 at homologous positions to those observed in PTBP1 (start-loss and p.Arg14Thr). Clinically, they exhibited developmental delay without skeletal dysplasia, consistent with mostly brain-restricted expression of PTBP2. Molecularly, the variants showed similar cytoplasmic retention and increased P-body localization. Transcriptomic profiling of fibroblasts, available from one individual, closely resembled that of PTBP1 start-loss cases, suggesting shared pathophysiological mechanisms. Further identification of PTBP2 cases will help distinguish gene-specific from common disease pathways across the polypyrimidine-tract protein family.

## Methods

### Sex as a biological variable.

Sex was not considered as a biological variable for genetic analysis and the human skin fibroblast transcriptomic and proteomic analyses.

### Statistics.

For the in vitro studies, data are expressed as the mean ± SD. Statistical analyses were performed using GraphPad Prism software (GraphPad Software) with a 2-tailed Student’s *t* test or Mann–Whitney U test for quantification, localization, colocalization, and Duolink spot counts. The frequencies of the phenotypes observed in mRNA-microinjected zebrafish embryos were analyzed with the χ^2^ test. A *P* value ≤ 0.05 was considered statistically significant.

For the proteomics analysis, raw MS data was processed using the MaxQuant software v2.1.4.0 (76). Peak lists were searched against a database including 40,992 human protein sequences extracted from SwissProt (16-10-2022) as well as common contaminants with reversed sequences. MaxQuant parameters were set as follows: MS tolerance at 20 ppm for the first search and 10 ppm for the main search, MS/MS tolerance at 40 ppm, maximum number of missed cleavages at 1, cysteine carbamidomethylation as a fixed modification, and methionine oxidation as a variable modification. FDRs were estimated based on the number of hits after searching a reverse database and were set at 1% at both peptide spectrum (with a minimum length of seven amino acids) and protein levels. Data normalization and protein quantification used the label-free quantification (LFQ) option implemented in MaxQuant with a “minimal ratio count” of one. The “match between runs” option was enabled using a 2-minute time window after retention time alignment. All other MaxQuant parameters were set as default. Statistical analysis was performed using the open-source ProStar software (77). After filtering proteins for their presence in all 3 replicates of at least 1 condition, missing values were imputed using the normal distribution. Data was then subjected to hypothesis testing using Limma’s test with FDR 0.03 using Benjamini–Hochberg correction. LFQ intensities of the 5% most variable protein values were used to draw the heatmap.

### Study approval.

Written informed consent was obtained from the individual(s) and minors’ legal guardian/next of kin for the publication of any potentially identifiable images or data included in this article. The study involving human participants was reviewed and approved by the appropriate institutional review board of Dijon University Hospital. The study was conducted within the framework of the DISCOVERY project (N° ID RCB: 2016-A01347-44 N° CPP EST I: 2016/38). Samples were part of the GAD collection DC2011-1332.

### Data availability.

The bulk and RIP-seq transcriptomic data have been deposited in the European Genome-Phenome Archive (EGA) under the study accession number EGAS50000001210. Supporting Data Values and files including further Methods are included in the Supplemental Material.

## Author contributions

LOF, QT, CM, DAK, TG, OM, VCD, PAT, NEV, ASP, MWW, AB, LMB, RSH, CTG, ACEH, ERR, MM, SEH, KO, RH, RBG, AR, DW, KP, JL, REM, A Costantini, HV, SR, GP, MF, RZ, JD, A Morgan, DJA, CTR, MAM, FL, GY, LAPJ, and CG conducted the patient clinical evaluations. QT, LOF, EC, OM, and VCD curated the clinical data. R Pfundt, EDB, LELMV, LAMA, TR, LCZ, SADM, MJGS, R Person, FT, MLT, SMH, ROL, JMH, KL, FTMT, CH, CTG, NE, NM, HS, MSH, DC, MT, CP, BS, JT, FB, CPR, MMA, and MSP interpreted the genome/exome data. A Masson performed the knockdown and transcriptomic experiments, bioinformatics analyses including differential expression, biological pathway enrichment and splicing analysis, transcriptomic and proteomic data analysis and visualization. JP performed the site-directed mutagenesis, transfection assays, cycloheximide chase assays, fluorescence and confocal imaging, immunostaining, Western blots, sequence alignment analysis, and quantified and analyzed associated data. M Orefice and M Ori conducted functional assays in zebrafish and analyzed associated data. JMS and CSR generated and analyzed the proteomic data. RR, SG, MAL, JK, and HM generated and interpreted the DNA methylation data. LDJ, FEI, SN, MC, and VB contributed to functional data generation. LDJ, A Masson, JP, FEI, DLB, and QT contributed to data interpretation. LDJ, JP, A Masson, NM, and PC managed cell cultures. YD, A Ciolfi, and VV contributed to the bioinformatic analysis. FT performed the bioinformatic analysis of whole-genome sequencing of Individuals 11, 12, and 13. A Masson, JP, M Ori, M Orefice, QT, and AV, wrote the manuscript. AV conceptualized the study, conceived the experiments, and reviewed the manuscript.

## Funding support

This work is the result of NIH funding, in whole or in part, and is subject to the NIH Public Access Policy. Through acceptance of this federal funding, the NIH has been given a right to make the work publicly available in PubMed Central.

- Dijon University Hospital.
- ISITE-BFC (PIA ANR).
- The European Union through the FEDER programs.
- The Priority Research Programme on Rare Diseases of the French Investments for the Future Programme, MultiOmixCare project.
- Italian Ministry of Health (PNRR-MR1-2022-12376811).
- Italian Ministry of Universities and Research (FOE 2020).
- European Union’s Horizon 2020 research and innovation program grant agreement number 779257.
- Genome Canada and the Ontario Genomics Institute (OGI-188).
- French Proteomic Infrastructure (ProFI) project (grant ANR-10-INBS-08 & ANR-24-INBS-0015).
- Sigrid Jusélius Foundation, Swedish Research Council (2022-00800).

## Supplementary Material

Supplemental data

Unedited blot and gel images

Supplemental table 1

Supplemental table 2

Supplemental table 3

Supplemental table 4

Supplemental table 5

Supplemental table 6

Supporting data values

## Version 1 09/18/2025

In-Press Preview

## Version 2 11/17/2025

Electronic publication
